# Molecular and Genetic Analysis of the Increased Number of Genes for *Trypanosoma cruzi* Microtubule Associated Proteins in the Class Kinetoplastida

**DOI:** 10.3390/pathogens14050476

**Published:** 2025-05-14

**Authors:** Martin A. Winkler, Alfred A. Pan

**Affiliations:** 1 Biotech Advisor, 1321 Wagon Wheel Road, Lawrence, KS 66049-3543, USA; 2 TNTC, Inc., 25A Crescent Drive, Pleasant Hill, CA 94523-5508, USA

**Keywords:** *Trypanosoma cruzi*, *Trypanosoma brucei*, antigen 36, Chagas disease, microtubule associated protein, genetic diversity, molecular bioinformatics

## Abstract

*Trypanosoma cruzi* GenBank^®^ M21331 encodes for Antigen 36 (Ag 36), which is a tandemly repeated *T. cruzi* antigen. GenBank M21331 has a gene sequence similarity to human immune genes IFN-α, IFN-β, and IFN-γ, as well as to human *TRIM* genes. A BLAST-p search revealed that *T. cruzi* GenBank M21331 had seven gene sequences homologous to microtubule-associated protein (MAP) genes with a 100% amino acid sequence identity. There are 36 genes in the *T. cruzi* genome with >94% identity to GenBank M21331, and these genes encode proteins ranging in size from 38 to 2011 amino acids in length, the largest containing 20, 25, and 30 repeats of the Ag 36 thirty-eight-amino-acid-sequence motif. The purpose of this study was to perform a genetic and molecular comparative analysis of *T. cruzi* GenBank M21331 to determine if this gene sequence is unique to the *T. cruzi* clade, present in the *T. brucei* clade, and/or exists in other trypanosomatids. There are seven homologous genes to GenBank M21331 in *T. cruzi*, but only one homolog found of this gene in *T. brucei*. The MAP genes in *T. cruzi* appear to have expanded at least eleven-fold in number compared to similar MAP genes in *T. brucei*. The DNA sequences and functions of these MAP genes in their respective species and clades will be discussed and are a fascinating area for further scientific study.

## 1. Introduction

Trypanosomatid parasites in the class Kinetoplastida include *Trypanosoma cruzi*, *T. brucei* ssp., and *Leishmania* spp., are the causative human agents of Chagas disease, African sleeping sickness, and Leishmaniasis, respectively [1]. The genome sequences of these three trypanosomatids, *T. cruzi*, *T. brucei*, and *L. major*, are now available to aid in genetic, immunological, and molecular research [2,3,4,5]. In addition, a whole genome assembly of a hybrid *Trypanosoma cruzi* strain has been assembled with Nanopore sequencing [6]. Investigations into the biological mechanisms of *T. cruzi* are perplexing due to the multifaceted nature and unique characteristics of its genes. *Trypanosoma cruzi* is diploid, containing homologous chromosome pairs which are differentially sized [7]. Its genome has been sequenced and is estimated to be between 106.4 and 110.7 Mb in size (diploid) [2,3]. At least 50% of the genome contains repetitive sequences in simple tandem repeats and consists of a large family of surface proteins, gene retrotransposons, and subtelomeric repeats. These repetitive genes may also be found as secreted proteins which serve to evade the host immune responses that are expressed concurrently [8,9,10,11]. In addition, *T. cruzi* genes encode numerous families of surface proteins (e.g., mucins and mucin-associated surface proteins) [12,13,14,15,16], and their genes also encode trans-sialidases [10,11,13,14,15,16,17,18,19]. It is hypothesized that this strategy may assist the amastigote form of *T. cruzi* to survive and propagate in susceptible hosts by evading recognition by T-cells [20,21].

*Trypanosoma cruzi* exemplifies a genetically diverse intra-species [22,23,24,25]. Based on analysis of genetic and biochemical indicators, there may be up to seven lineages (Typing Units) of TcI–TcVI [26,27,28,29,30,31,32] and TcBat [33]. However, there is immunological evidence that Chagas disease may also be due to a single parasite lineage based on a small surface molecule [34]. These lineages may produce distinct pathological manifestations ranging from CCC (Chronic Chagas Cardiomyopathy) in endemic areas to enlargement of the esophagus and colon (known as mega syndromes). Typing Unit TcI (e.g., Sylvio X10/1 and Dm28c [35,36] and Dm25 [13]) belong to Typing Unit TcI, a group related to the sylvatic cycle that is associated with human disease in endemic countries north of the Amazon basin [36,37,38,39,40], and is also found in the transmission cycle between insect vector and animals. Typing Unit TcII mainly occurs in domestic settings in the southern countries of South America [36,38,40,41] and causes tissue damage of the internal organs and digestive tract [39,40,42]. These Typing Units could be used as a target for drug discovery and reverse genetics [43].

In 2005–2006, a resurgence in neglected diseases arose [44,45,46,47,48,49,50], and Chagas disease has now been referenced as “the most neglected of the neglected diseases” [50]. The recognition of these impactful but neglected parasitic diseases has resulted in increased research in the areas of immunology, molecular biology, and genetics being used to study and characterize host–parasite relationships, and to identify antigenic molecules which are involved in mounting a protective immune response. Today, an estimated 6 to 7 million human individuals and countless susceptible mammalian species are infected with this parasite. It has been estimated that an additional 75 million people may be at risk of infection [51] leading to approximately 12,000 deaths every year. However, at present, no vaccine has been approved for these diseases and available drugs are highly toxic, with severe and frequent side effects, and may only be effective in combating circulating forms of the parasite [52]. In addition, the occurrence of drug resistance is also a possibility. Therefore, there is an urgent necessity to identify gene, protein, and carbohydrate targets, and understand their mechanisms of action(s). These may result in unique and specific markers for vaccines, as well as in the development of therapeutics. There is also the need for the development of highly sensitive and specific analytical diagnostic assays in blood and tissue specimens to combat further spread of these diseases [53,54,55,56].

In earlier investigations of diagnostic antigens, we identified one cloned gene from *T. cruzi* (Brazil strain) amastigotes from axenic culture [57] as a potential candidate. These axenic amastigotes have been shown to be comparable to amastigotes in cell culture (MRC-5 and Vero cells) [17]. This gene was sequenced and found to be identical to the repetitive antigen Clone 36, “Antigen 36” [9,58,59,60], and was also described as JL9 Antigen [61]. An initial search of the Wisconsin Package [62] with our DNA sequence disclosed similarity to human Ro52 with the translated sequence in the second reading frame of Ag 36. Direct comparison of the Ag 36 DNA sequence with the Ro52 DNA sequence revealed a 70% identity in one sequence of 44 nucleotides between the Ag 36 DNA sequence and *TRIM21*, the gene for human Ro52 [58]. Once the function of *TRIM21* was identified, we proposed that there may be a link between it and the gene for Ag 36 identified in CCC [58]. Ro52 is expressed in the immune system as a predominantly cytoplasmic protein that can be upregulated and translocated to the nucleus in a pro-inflammatory environment. A study was also conducted to compare *TRIM21* region sequences among mammalian species to the human *TRIM21* region to evaluate any similarities in non-human genes. Results indicated that related sequences were present in 11 mammalian species [60]. Additionally, a BLAST-p search was conducted with GenBank^®^ M21331 against the *T. cruzi* genome to determine the minimum number of genes coding for proteins closely related to Ag 36. The BLAST-p revealed 7 unique GenBank accession entries which produced seven proteins 100% identical to Ag 36 of the 14 GenBank entries previously reported [60]. We have also shown that GenBank M21331 has a significantly similar gene sequence identity to human immune genes (IFN-α, IFN-β, and IFN-γ) and to human *TRIM* genes, such as *TRIM40* and *TRIM21* [60]. Those results appeared to be the first description of molecular mimicry of immune genes in humans by a protozoan parasite [60]. The protein generated from this gene has also been used in the development and implementation of a diagnostic assay [63,64,65].

A phylogenetic tree for these trypanosomatids was developed by Stevens [1], and indicated a *T. cruzi* clade, a *T. brucei* clade, and an aquatic clade. However, unfortunately no data are available in GenBank on the aquatic clade to perform a genetic comparison. Additional evolutionary history on *T. cruzi* is provided by Maslov [19], Briones [66], and Rozas [67]. In this study, we focus on a genetic and molecular comparative analysis of *T. cruzi* GenBank M21331 to organisms in the class Kinetoplastida to determine if this gene sequence is exclusive to members in the *T. cruzi* clade or is present in the *T. brucei* clade. Microtubule associated proteins are present in the *T. cruzi* clade, as designated above, and have also been described in trypanosomes present on the African continent, such as MARP-1 (a repetitive non-variable antigen) [68,69] that is localized on the microtubules of the parasite’s cell body and flagellum. MARP-1 comprises 50 repeats of a 38 amino acid motif in *T. b. brucei* and *T. b. gambiense* [70,71]. In addition, MAPs are present in other African trypanosomes such as *T. vivax*, *T. congolense*, *T. evansi*, and *T. equiperdum*, as well as New World trypanosomes, such as *T. rangeli* and *T. theileri*, and in *Leishmania* spp.

The Kinetoplastid groups of parasites diverged approximately 500 million years ago in different habitats worldwide [1,72]. It is also speculated that *T. cruzi* arose over 150 million years ago, infecting animals throughout Laurasia and Gondwanaland, which are the regions that eventually formed North and South America, respectively [66]. It is theorized that the disease occurred in humans approximately 15,000–20,000 years ago in the late Pleistocene era when they were migrating into these areas. These trypanosomatids have thus genetically evolved over millennia to each develop unique molecular mechanisms to evade destruction from the innate immune system of the host. GenBank M21331 and related genes in American and African trypanosomes are conserved as MAPs and can be used as genetic, immunological, and molecular biomarkers. The MAP genes, the DNA sequences, and their functional role in trypanosomes and in their respective species and clades are intriguing, and they will be further evaluated, investigated, and analyzed.

## 2. Materials and Methods

### 2.1. Cloning of Trypanosoma cruzi Amastigote Genes

We identified one cloned gene from *Trypanosoma. cruzi* (Brazil strain) amastigotes grown in axenic culture [57], characterized it [17], sequenced it by the Sanger method [73], and found it to be identical to the repetitive antigen Clone 36, “Antigen 36” [9,58,59,60].

### 2.2. BLAST-p Search to Determine Number of Ag 36 Homologues

The GenBank M21331 gene was translated into its amino acid sequence using the translation tool at https://usegalaxy.org (accessed on 25 March 2025) [74,75] and the sequence entered in the BLAST-p search box to determine homologous genes in the *T. cruzi* genome. The BLAST-p search algorithm was selected at https://blast.ncbi.nlm.nih.gov/Blast.cgi (accessed on 25 March 2025). The search entered into the database read *Trypanosoma cruzi* taxid 5693, or *Trypanosoma cruzi* Dm28c taxid 1416333, and the first 100 most homologous genes (in order of their homology to GenBank M21331) and their amino acid sequences were downloaded and saved as a text file. The “e value” noted in Table 1 is the probability that this result happened by random chance.

### 2.3. BLAST-p Search of Trypanosoma brucei with Trypanosoma cruzi GenBank M21331

The GenBank M21331 gene was translated into its amino acid sequence using the translation tool at https://usegalaxy.org [74,75] and the sequence was entered in the BLAST-p search box to determine homologous genes in the *T. brucei* genome. The BLAST-p search algorithm was selected at https://blast.ncbi.nlm.nih.gov/Blast.cgi. The search entered into the database read *Trypanosoma brucei* taxid id 5691, and the first 100 most homologous genes (in order of their homology to GenBank M21331) and their amino acid sequences were downloaded and saved as a text file.

### 2.4. BLAST-p Search of Leishmania donovani with Trypanosoma cruzi GenBank M21331

The GenBank M21331 gene was translated into its amino acid sequence using the translation tool at https://usegalaxy.org [74,75] and the sequence was entered in the BLAST-p search box to determine homologous genes in the *L. donovani* genome. The BLAST-p search algorithm was selected at https://blast.ncbi.nlm.nih.gov/Blast.cgi. The search entered into the database read *Leishmania donovani* taxid id 5661, and the first 100 most homologous genes (in order of their homology to GenBank M21331) and their amino acid sequences were downloaded and saved as a text file.

## 3. Results

To determine the number of homologues of Ag 36 in the *T*. *cruzi* genome, a BLAST-p search of the https://blast.ncbi.nlm.nih.gov/Blast.cgi database with the Ag 36 protein sequence was conducted. It revealed 7 unique GenBank accession entries which produced seven proteins 100% identical to Ag 36 of the 14 GenBank entries previously reported [60], as shown in Table 1. There were no genes found with the BLAST-p of Dm28c [35] and other single genomes. Sylvio X10/1 [35,36] and Dm25 [13] were not available for a BLAST-p search in the NCBI database.

**Figure 1:**
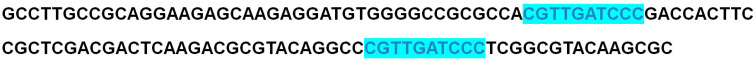
*Trypanosoma cruzi* antigen DNA (ENA|M21331) Ag 36 from GenBank. The internal ten-nucleotide-duplicated sequences are highlighted in blue and in blue face font.

**Figure 2:**
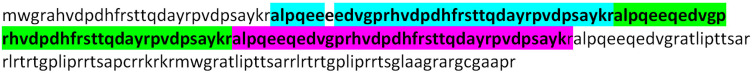
Example of an amino acid sequence comparison of *Trypanosoma cruzi* (GenBank PWU83737) with Ag 36. The Ag 36 amino acid sequences, repeated three times, are shown in bold face font and highlighted in blue, green and purple. Note that there is one amino acid substitution (see between the blue highlighted area, seventh nucleotide).

In addition, following the search of the *T. cruzi* genome at https://www.ncbi.nlm.nih.gov/, there were 36 genes with greater than 94% identity to GenBank M21331 (Table 2), with 35 proteins containing multiple copies of the Ag 36 sequence motif. These genes ranged from 38 to 2011 amino acids in length, as shown in Table 2. For each entry in Table 2, the number of repeats of the Ag 36 motif (identified as homologous domains by the BLAST-p search program) is shown in Table 3. There are repeats of 1–10, 11–12, 17, 19, 20, and 25 copies, and two entries have a maximum of 30 repeats. In the 36 entries shown in Table 3, there are a total of 296 total copies of the Ag 36 protein sequence motif. The high number of genes with multiple copies of the Ag 36 sequence may be due to the internal sequence duplication in the gene of Ag 36, GenBank M21331. The gene sequence coding for Ag 36 M21331 was obtained from its GenBank entry in FASTA format to indicate an internal duplication (Figure 1). The ten-nucleotide sequence is shown in blue faced font and highlighted twice is an internal duplication found in this gene and in its homologues. This internal sequence duplication may have increased the chance of unequal crossing over between two copies of the gene during meiosis, producing larger, duplicated genes [76]. It is of interest that meiosis has been reported in *T. cruzi* [77,78]. Figure 2 shows an example of a *T. cruzi* gene, GenBank PWU83737, with multiple (3) copies of the Ag 36 amino acid sequence.

This result allowed us to further explore the similarity of GenBank M21331 to genes in other species in the class Kinetoplastida MAPs. BLAST-p searches of Ag 36 amino acid sequences were also performed versus genomes of *T. brucei* (ssp.), *T. congolense* and *L. donovani*, *T. theileri*, and *T. vivax*. The resulting matches were ranked by percent identity to Ag 36 sequence and the highest percent identities are shown in Table 4. The highest percent identities are, therefore, most directly related to the Ag 36 sequence.

## 4. Discussion

The purpose of this study was to compare, enumerate, and analyze DNA sequences between *T. cruzi* GenBank M21331, which codes for Ag 36, and similar genes found in the class Kinetoplastida. We focused on a genetic and molecular comparative analysis of *T. cruzi* GenBank M21331 (Ag 36) to determine if this gene sequence is exclusive to members in the *T. cruzi* clade, or present in the *T. brucei* clade, and/or other trypanosomatids. A BLAST-p search of Ag 36 protein, versus the translated *T. cruzi* genome at the NCBI website, disclosed that there are 43 *T. cruzi* gene products (seven homologues and 36 genes containing multiple copies) that are homologous in protein sequence to Ag 36, implying that there are 43 genes that are very similar or identical to GenBank M21331 in *T. cruzi.* Thirty-six of the protein matches were greater in length than Ag 36 and contained one or more sequences homologous or partially homologous to Ag 36. A BLAST-p search of Ag 36 was performed on *T*. *theileri*, *T. vivax*, *T. brucei*, *T. b. brucei*, *T. b. gambiense T. congolense*, and *Leishmania* spp. genomes. *Trypanosoma theileri* and *T. vivax* showed 83 and 57% identities, in line with their close phylogenetic relationship to *T. cruzi*. Similarly, *T. brucei*, *T. b. brucei*, and *T. b. gambiense* showed proteins 69% homologous to Ag 36. Additionally, *T. b. equiperdum* and *T. congolense* had proteins 65% homologous to Ag 36, and *L. donovani. Leishmania tropica* and *L. mexicana* had proteins 47% homologous to Ag 36 (Table 4). These results show that there is an eleven-fold greater number of copies of the MAP genes related to GenBank M21331 in *T. cruzi* compared to *T. brucei* spp., from the following calculation: Fold of *T. cruzi* compared to *T. brucei* spp. = (7 *T. cruzi* homologues + 36 closely related genes)/(4 *T. brucei* spp. Genes) = 43/4 = 10.75. *Trypanosoma cruzi* strain Dm28c [35] provided no BLAST-p results, which implies that this strain and perhaps others in Typing Unit TcI do not have this gene and its homologues. Sylvio X10/1 [36], Dm25c [13], and other individual strains were not available at NCBI to be searched by a BLAST-p as a genome.

The specific and definitive roles these *T. cruzi* MAPs may play, besides their possible association with microtubules, is yet to be defined. However, based on the similarity of GenBank M21331 to IFNs, *TRIM21* and other *TRIM* (tripartite motif) genes, GenBank M21331 mRNAs may play a crucial part in suppressing host mRNA translation of the IFN and *TRIM* genes that are involved in the innate immune response to *T. cruzi* [59,60]. *Trypanosoma theileri*, a closely related species to *T. cruzi* in the Kinetoplast phylogeny [1], showed a gene 83% identical to GenBank M21331. The most identical gene in *T. theileri* did not show the internal ten nucleotide repeat found in GenBank M21331 (excluding the overall repeats due to it being a repetitive antigen). There were no additional larger homologues of the *T. theileri* gene as were observed in *T. cruzi* with GenBank M21331. This finding supports the theory that the internal repeat in GenBank M21331 may have given rise to the additional larger homologues. *Trypanosoma theileri* is a blood borne trypanosome of cattle commonly transmitted by biting flies. It does not typically invade and does not reside inside of cells as *T. cruzi* does. *Trypanosoma cruzi* has an eleven-fold greater number of GenBank M21331 MAPs compared to *T*. *brucei* spp., which do not have an intracellular stage. *Trypanosoma vivax*, another parasitic disease of cattle and wild mammals that survives extracellularly in its host, disclosed three genes (986, 1318, and 2957 nucleotides in length) with 57% identity which were much larger when compared to GenBank M21331. The results in this study indicate that there are at least an eleven-fold greater number of copies of MAP genes related to GenBank M21331 in *T. cruzi* compared to *T. brucei* spp.

There may be up to seven lineages of *T. cruzi* from analysis of genetic, molecular, and biochemical indicators [22,23,24,25,26,27,30,31,32,33,34,35,36,37,39,41,42,43]. It is surprising that GenBank M21331 and its family of 43 homologous genes were not found in *T. cruzi* strain Dm28c [35], representative of Typing Unit TcI (sylvatic cycle). Either the genes related to GenBank M21331 were lost from this strain, or never gained them as in Typing Units TcII, III, V, and VI. The divergence between these genes in the typing groups may be a result of meiosis, which has been reported in *T. cruzi* [77,78], with genetic exchange occurring between lineages. Therefore, divergence can occur due to recombination between organisms once thought to reproduce asexually. However, due to biomarker and sequence analysis of conserved genes, a common genetic ancestor may eventually be identified.

The bioinformatics tools such as BLAST-p that have been utilized in this study have been shown to be powerful, and have been used extensively for investigations in immunology, infectious disease, drug discovery, and other areas of biomedical research [80]. In combination with recent genomic data, we have used bioinformatics to trace the phylogeny of genes related to GenBank M21331 in Trypanosomes, resulting in an eleven-fold greater number in most lineages of *T. cruzi* compared with *T*. *brucei*. The seven Typing Units of *T. cruzi*, TcI through TcVII and TcBat, have been associated by their epidemiology and ecological scenarios [13,81]. They can be distinguished in the laboratory by analysis of their kinetoplast mini-circles, nuclear DNA, and other genetic or electrophoretic markers [37,38,82,83]. Experiments can be designed to test the Ag 36 family of genes as a new marker to discriminate TcI from the other Typing Units. The GenBank M21331 gene has apparently been duplicated to form a family of 36 related genes in all Typing Units except for TcI. The absence of these genes in TcI is confirmed by negative immunoassay results for Ag 36 in TcI serum samples from Chagas patients, along with positive immunoassay results in the other Typing Units [63,64]. The Ag 36 family of genes, along with the Ag 36 serology, can be further used in laboratory experiments to compare infectivity and disease severity between Typing Units.

The Kinetoplastid groups of parasites diverged approximately 500 million years ago in different parts of the world [1,72]. *Trypanosoma cruzi* arose over 150 million years ago, infecting animals in North and South America [66]. GenBank M21331 and related genes are conserved as MAPs between American and African trypanosomes and can be useful as genetic, immunological, and molecular biomarkers. The *T. cruzi* MAP genes partial identities to human immune genes (IFN-α, IFN-β, IFN-γ, and human *TRIM* genes) may be relevant to the parasite’s resistance to the mammalian innate immune system during infection. Further experiments can be designed [60] to test GenBank M21331 and related genes for their role in resistance to innate immunity.

## Figures and Tables

**Table 1:** Output matrix resulting from the BLAST-p search of Ag 36 amino acid sequence versus the *Trypanosoma cruzi* genome, performed at and retrieved from https://www.ncbi.nlm.nih.gov/ (accessed on 25 March 2025).

GenBank Accession Number	% Identity	Length (Amino Acids)	Mismatches (Nucleotides)	Amino Acid Residues Start	Amino Scid Residues End	e Value *	% Positives
RNC30406	97.368	38	1	111	148	4.14 × 10^−17^	100
PWU97874	97.368	38	1	107	144	8.82 × 10^−17^	100
KAF8288323	97.222	36	3	33	68	2.35 × 10^−15^	100
KAF8288323	97.368	38	1	145	182	1.89 × 10^−16^	100
KAF8288323	96.667	30	9	1	30	4.22 × 10^−11^	100
PWU84425	97.368	38	1	328	365	1.94 × 10^−16^	100
PWV17283	97.368	38	1	182	219	3.99 × 10^−16^	100
PWU83738	97.368	38	1	107	144	5.01 × 10^−16^	100
PWU83738	97.368	38	1	335	372	5.01 × 10^−16^	100
PWU83738	97.368	38	1	373	410	5.01 × 10^−16^	100
PWU83738	97.368	38	1	411	448	5.01 × 10^−16^	100
PWU83738	97.368	38	1	449	486	5.01 × 10^−16^	100
PWU83738	97.368	38	1	487	524	5.01 × 10^−16^	100
XP_809567	97.297	37	1	41	77	8.11 × 10^−15^	100

* The “e value” is the probability that this result happened by random chance.

**Table 2:** Overall results of the BLAST-p search of Ag 36 amino acid sequence versus the *Trypanosoma cruzi* genome showing multiple copies of the Ag 36 protein sequence, performed at and retrieved from https://www.ncbi.nlm.nih.gov/. Accession identifiers are at GenBank Gene https://www.ncbi.nlm.nih.gov/gene/.

Description	e Value *	Percent Identity	Amino Acid Length	GenBank Accession ID
microtubule associated protein homolog	2.00 × 10^−20^	94.59	38	AAB20531
microtubule-associated protein	5.00 × 10^−21^	100	103	RNC30144
putative microtubule-associated protein	4.00 × 10^−21^	100	116	KAF8291685
putative microtubule-associated protein	1.00 × 10^−20^	97.37	121	KAF8288266
microtubule-associated protein	5.00 × 10^−20^	97.37	142	RNF14378
putative microtubule-associated protein	8.00 × 10^−21^	100	157	PWV17285
hypothetical protein TcYC6_0124180	3.00 × 10^−20^	100	159	KAF8291204
hypothetical protein TcBrA4_0014660	6.00 × 10^−20^	97.37	159	KAF8288041
microtubule-associated protein	3.00 × 10^−19^	97.37	166	RNC29983
microtubule-associated protein	1.00 × 10^−19^	97.37	170	RNC47282
putative microtubule-associated protein	8.00 × 10^−20^	97.37	173	KAF8288373
putative microtubule-associated protein	1.00 × 10^−19^	97.37	195	KAF8287749
microtubule-associated protein	5.00 × 10^−18^	100	227	RNC30406
putative microtubule-associated protein	4.00 × 10^−20^	100	233	PWV17284
putative microtubule-associated protein	4.00 × 10^−20^	100	234	PWU83737
microtubule-associated protein-like	1.00 × 10^−19^	100	235	KAF8291360
microtubule-associated protein	6.00 × 10^−14^	97.06	240	RNC30522
MAP-TcD-TSSA-FRA-SAPA chimeric antigen	3.00 × 10^−13^	100	266	UGO57631
microtubule-associated protein homolog	8.00 × 10^−19^	97.37	299	AAD51095
hypothetical protein TcBrA4_0014630	3.00 × 10^−19^	97.37	310	KAF8288323
microtubule-associated protein-like	3.00 × 10^−19^	100	311	KAF8291458
microtubule-associated protein	3.00 × 10^−20^	100	321	RNC47283
microtubule-associated protein-like	5.00 × 10^−19^	100	363	KAF8291386
putative microtubule-associated protein	1.00 × 10^−10^	100	385	PWV17283
microtubule-associated-like protein	4.00 × 10^−19^	100	391	KAF8288016
hypothetical protein TcBrA4_0014640	2.00 × 10^−18^	97.37	441	KAF8288063
microtubule-associated protein	6.00 × 10^−19^	100	555	KAF8291499
putative microtubule-associated protein	6.00 × 10^−21^	97.37	576	PWU83738
putative microtubule-associated protein	2.00 × 10^−20^	100	644	PWU84425
putative microtubule-associated protein	6.00 × 10^−20^	100	652	PWU83735
microtubule-associated protein, putative	6.00 × 10^−19^	100	738	XP_803031
putative microtubule-associated protein	7.00 × 10^−19^	100	990	KAF8291180
microtubule-associated protein, putative	4.00 × 10^−19^	100	1091	XP_809567
putative microtubule-associated protein	3.00 × 10^−19^	100	1122	PWV17287
putative microtubule-associated protein	6.00 × 10^−19^	100	1180	PWU97875
putative microtubule-associated protein	4.00 × 10^−20^	100	2011	PWU97874

Amino Acid Length = Total amino acids in the protein; HP = Hypothetical Protein [79]; MAP = Microtubule Associated Protein Percent Identity = Percent exact matches in sequence; pMAP = putative Microtubule Associated Protein. * The “e value” is the probability that this result happened by random chance.

**Table 3:** Copies of the Ag 36 amino acid sequence motif reported in the BLAST-p genes of Table 2.

Accession GenBank	Ag 36 Motif Copies	Accession GenBank	Ag 36 Motif Copies
AAB20531	1	KAF8288323	6
RNC30144	3	KAF8291458	8
KAF8291685	3	RNC47283	17
KAF8288266	2	KAF8291386	4
RNF14378	1	PWV17283	30
PWV17285	5	KAF8288016	5
KAF8291204	4	KAF8288063	11
KAF8288041	2	KAF8291499	12
RNC29983	4	PWU83738	7
RNC47282	17	PWU84425	9
KAF8288373	2	PWU83735	1
KAF8287749	1	XP_803031	19
RNC30406	1	KAF8291180	25
PWV17284	1	XP_809567	20
PWU83737	3	PWV17287	20
KAF8291360	11	PWU97875	30
RNC30522	0	PWU97874	17
UGO57631	12	**Copies Sum**	296
AAD51095	1		

GenBank Accessions are the genes listed from Table 2. Ag 36 motif copies are the number of copies of the Ag 36 amino acid sequences in the translated gene, as reported in the BLAST-p search of Ag 36 on the *T*. *cruzi* genome.

**Table 4:** Comparison in a BLAST-p search of Ag 36 in the class Kinetoplastida.

Description	Organism	Percent Identity	Accession Length Amino Acid Residues
MAP	*Trypanosoma theileri*	83	133
MAP [68]	*T. brucei*	69	145
MAP [68]	*T. brucei*	69	313
pMAP	*T. b. brucei* TREU927	69	2105
HP Tb10.v4.0053	*T. b. brucei* TREU927	69	4119
MAP 2	*T. b. brucei* TREU927	69	4880
pMAP	*T. b. brucei* TREU927	69	2257
pMAP	*T. b. gambiense* DAL972	69	1687
pMAP, (fragment)	*T. b. gambiense* DAL972	69	2245
HP, unlikely	*T. b. gambiense* DAL972	65	416
MAP 2	*T. b. equiperdum*	65	679
UPP	*T. congolense* IL3000	65	440
MAP MARP-1 [70]	*T. brucei*	58	192
HP, unlikely	*T. b. gambiense* DAL972	58	725
MAP	*Trypanosoma vivax*	57	1318
MAP P320	*T. b. brucei*	48	290
HP	*Leishmania donovani* *	47	1124

Accession Length = total amino acids in the protein; HP = Hypothetical Protein [79]; MAP = Microtubule Associated Protein; MARP-1 = Microtubule Associated Repetitive Protein; pMAP = putative Microtubule Associated Protein; Percent Identity = percent exact matches in sequence; UPP = Unnamed Protein Product; BLAST-p search accomplished at http://ncbi.nlm.nih.gov/. * BLAST-p search performed on *L. tropica* and *L. mexicana* were similar to *L. donovani* with proteins that were 47% identical.

## Data Availability

The datasets generated during and/or analyzed during the current study are available in the online Galaxy repositories: https://usegalaxy.eu/u/martinawinklerphd/h/copy-of-copy-of-mammalian-trim-genes-compared-with-antigen-36 (accessed on 24 April 2025); https://usegalaxy.org/u/martinawinklerphd/h/ag36-and-mammalian-trim21-homologies (accessed on 24 April 2025). These bioinformatics workflows are available to all, but registration (which is free) is required to view the results.

## Abbreviations

The following abbreviations and definitions are used in this manuscript: Antigen 36 (Ag 36)—(clone A2; clone 36; Tc36; JL9)—The tandemly repeated *T. cruzi* antigen reported by Ibañez [9], Levin [61], and Winkler [58,59,60], which is highly reactive with Chagasic sera [63,64]. GenBank M21331—GenBank^®^ is a database that contains publicly available nucleotide sequences for genus/species organisms. The library is obtained through submissions from laboratories and batch submissions from large-scale sequencing projects. *T. cruzi* GenBank M21331 encodes for Antigen 36 (Ag 36). MAP (Microtubule Associated Proteins)—Microtubule associated proteins regulate assembly and stability of microtubules. Microtubules constitute a major part of the cytoskeleton and are important in cytoskeletal rearrangements during neuronal growth, axon guidance, and synapse formation. The Ag 36 gene of *T. cruzi* has seven homologous genes sequences (MAP genes with 100% amino acid sequence identity) to GenBank M21331. MARP-1 (Microtubule-Associated Repetitive Proteins)—The microtubular membrane framework of *T. brucei* containing two closely related, repetitive, high-molecular-weight MAPs, MARP-1 and MARP-2. The structure consists of a 38-amino-acid repeat over an approximate length of about 320 kDa, which are tandemly arranged and conserved.
